# Integrated microenvironment‐associated genomic profiles identify LRRC15 mediating recurrent glioblastoma‐associated macrophages infiltration

**DOI:** 10.1111/jcmm.16563

**Published:** 2021-05-07

**Authors:** Haichao Tang, Wensi Liu, Zhaoxu Xu, Jianhang Zhao, Weitao Wang, Zhaojin Yu, Minjie Wei

**Affiliations:** ^1^ Department of Pharmacology School of Pharmacy China Medical University Shenyang China; ^2^ Liaoning Key Laboratory of molecular targeted anti‐tumor drug development and evaluation Liaoning Cancer immune peptide drug Engineering Technology Research Center; Key Laboratory of Precision Diagnosis and Treatment of Gastrointestinal Tumours, Ministry of Education, China Medical University Shenyang China

**Keywords:** immunology, LRRC15, macrophages, recurrent glioblastoma, tumour microenvironment

## Abstract

Glioblastoma (GBM) is the most common malignant intracranial tumour with intrinsic infiltrative characteristics, which could lead to most patients eventually relapse. The prognosis of recurrent GBM patients remains unsatisfactory. Cancer cell infiltration and their interaction with the tumour microenvironment (TME) could promote tumour recurrence and treatment resistance. In our study, we aimed to identify potential tumour target correlated with rGBM microenvironment based on the gene expression profiles and clinical information of rGBM patients from The Cancer Genome Atlas (TCGA) database. LRRC15 gene with prognostic value was screened by univariate and multivariate analysis, and the correlation between macrophages and LRRC15 was identified as well. Furthermore, the prognosis correlation and immune characteristics of LRRC15 were validated using the Chinese Glioma Genome Atlas (CGGA) database and our clinical tissues by immunochemistry assay. Additionally, we utilized the transwell assay and carboxy fluorescein succinimidyl ester (CFSE) tracking to further confirm the effects of LRRC15 on attracting microglia/macrophages and tumour cell proliferation in the TME. Gene profiles‐based rGBM microenvironment identified that LRRC15 could act in collusion with microglia/macrophages in the rGBM microenvironment to promote the poor prognosis, especially in mesenchymal subtype, indicating the strategies of targeting LRRC15 to improve macrophages‐based immunosuppressive effects could be promising for rGBM treatments.

## 1 INTRODUCTION

Glioblastoma (GBM) is the most frequent malignant intracranial tumour, accounting for 47.1% of all malignant central nervous system tumours,^1^ and remains poor survival. The estimated median overall survival in primary GBM is 14.6 mo, and the two‐year survival rate is 26.5% after surgery and radiotherapy combined temozolomide.^2^ Furthermore, GBM nearly irresistible relapse because of intrinsic infiltrative characteristics. The median overall survival is about 24‐44 wk of recurrent GBM (rGBM).^3^ Currently, most studies mainly focussed on the primary and untreated tumours, while the biology and treatment design of rGBM remains challenging. Additionally, the current treatments could not effectively eradicate infiltrative tumour cells to reverse recurrence of GBM and could induce tumour cells to produce therapy‐resistance.^4^ Therefore, novel treatment strategies for GBM, especially for rGBM, are urgently needed.

Previous studies indicated that cancer cell proliferation and their interaction with the tumour microenvironment (TME) contributed to tumour recurrence and treatment resistance.^5^ GBM microenvironment is full of multiple immunosuppressive mechanisms, including the secretion of immune suppressive factors, the up‐regulation of suppressive factors on the cell surface, the abatement of T cells and NK cells, and the expansion of immune suppressive cells.^6^ The current immunotherapeutic approaches mainly focus on the checkpoint blockades with CD8^+^ T cells activation effects, such as Checkmate 143 (NCT02017717), whereas which did not get decent results.^7^, ^8^ Therefore, concentrating on disrupting the immunosuppressive barrier directly could be a promising window for GBM treatments. Glioblastoma‐associated macrophages (GAMs) are the most dominant immune suppressive cells in the GBM microenvironment,^9^ which are derived from brain‐resident microglia/monocytes and peripheral monocytes/macrophages that tend to develop into the M2‐like phenotype in the GBM microenvironment.^10^ Meng et al suggested M2‐like macrophages could contribute to radiotherapy resistance,^11^ indicating eradiating macrophages effects immunotherapy could play a radiosensitizing role. Similarly, M2‐like macrophages could generate reactive oxygen and nitrogen intermediates to limit the effectiveness of chemotherapy and targeted molecular therapies.^12^, ^13^ In particular, the mesenchymal subtype with more degree malignancy in rGBM was associated with limited clinical response and radioresistance,^14^ largely because of the infiltration of GAMs as well.^15^, ^16^ Therefore, impeding GAMs recruitment into the microenvironment could be a promising treatment strategy for improving the prognosis of rGBM.

The type I transmembrane protein 15‐leucine‐rich repeat containing membrane protein (LRRC15) is a member of the LRR superfamily that is thought to be involved in protein‐protein and protein‐matrix interactions, and signal transduction for various cellular processes.^17^, ^18^, ^19^ Previous studies demonstrated LRRC15 was highly expressed on cancer cells of mesenchymal origin with low normal tissue expression.^20^ However, the role of LRRC15 in rGBM has not been delineated.

In this study, we explored the microenvironment‐associated genetic landscapes in rGBM based on Estimation of Stromal and Immune cells in Malignant Tumor tissues using Expression (ESTIMATE) data through The Cancer Genome Atlas (TCGA) database. LRRC15 and its role in GAMs infiltration were identified. Besides, our study further integrated Chinese Glioma Genome Atlas (CGGA) data sets^21^, ^22^ and experimental methods to comprehensively confirm the prognostic value of LRRC15 and relationship between GAMs infiltration and LRRC15 expression.

## 2 METHODS AND MATERIALS

### 2.1 Gene expression database

The Level 3 gene expression profiles of rGBM patients were obtained on 30 March, 2019 from the TCGA data portal (https://tcga‐data.nci.nih.gov/tcga/). Clinical data, including subtypes and survival time, were also obtained from TCGA data portal. All gene expression were used as RNA‐seq by expectation‐maximization (RSEM). Level 3 gene expression profiles and clinical data of rGBM patients were downloaded on 6 May, 2020 from the Chinese Glioma Genome Atlas (CGGA) data set ( mRNAseq_693,^21^, ^22^ Illumina HiSeq and mRNAseq_325,^23^, ^24^ Illumina HiSeq 2,000 or 2,500) as validation (http://www.cgga.org.cn/). The mRNAseq_693 data set contained 109 rGBM samples. The mRNAseq_325 data set contained 24 rGBM samples.

### 2.2 Analysis of genes

Immune scores and stromal scores were calculated based on the ESTIMATE algorithm.^25^ All RNA expression data were divided into high/low immune score groups and high/low stromal score groups. Fold change >1.5 and *adj*. *P* < 0.05 were set as the cut‐offs to mine differential genes. Also, differential analysis of recurrent and primary GBM was performed with package edgeR. False‐positive discovery (FDR) < 0.05 and |log2 (fold change)| >1 were set as the cut‐offs to obtain differentially expressed genes (DEGs). The heatmaps and volcano maps were generated with R software.

Kaplan‐Meier plots were used to illustrate the correlation between patients’ overall survival and differential gene expression levels. The correlation was tested by log‐rank test. Univariate and multivariate analysis of key genes were further conducted using Cox progression.

Gene Ontology (GO) analysis was performed to identify the potential function of intersection genes using the clusterProfiler package in R software, and we were only interested in the significance level (*P* < 0.05 and enrichment >2).

Gene Set Enrichment Analysis (GSEA) between LRRC15 high expression and low expression was performed using GSEA 3.0. (https://www.gsea‐msigdb.org/gsea/index.jsp). The macrophages metagenes of GSEA were referenced based on the published study.^26^

LRRC15 expression in multiple cells was analysed using Human Protein Atlas (https://www.proteinatlas.org).

### 2.3 Immunohistochemistry assay

The 42 cases of human rGBM tissue samples as paraffin tissues microarrays and corresponding clinical data were obtained from the Fourth hospital of China medical university between January 2017 and March 2019. LRRC15 was evaluated using primary antibody (Anti‐LRRC15 antibody, 1:50, NPB1‐93556, Novus), and CD206 was evaluated using primary antibody (Anti‐Mannose antibody, 1:100, ab64693, abcam). The secondary antibody (Goat anti‐rabbit IgG, 1:50, ZDR‐5306; Zhongshan Gold Bridge Biotechnology, China) was used and the signal was detected using the DAB Kit (ZLI‐9018; Zhongshan Gold Bridge Biotechnology, China). The LRRC15 and CD206 expression were evaluated by two experienced researchers. The immunohistochemical staining intensity was scored on a scale of 0‐3 ( 0, no stain; 1, weak stain, 2, medium stain; 3, strong stain). The percentage of positive staining cells (0 to 100%) multiplied the score of immunohistochemical staining intensity (0‐3) equals the IHC scoring.

### 2.4 Cell culture and generation of THP‐1‐derived M2‐like macrophages

U‐87 MG cell lines were cultured in Dulbecco's Modified Eagle's Medium (DMEM, Gibco, 12 800 017) containing 10% FBS. Human monocyte cell lines (THP‐1) were cultured in RPMI‐1640 medium (Gibco, 31 800 022) with 10% FBS. All cells were cultured at 37°C with 5% CO_2_. THP‐1 cells were purchased from the Cell Resource Center of the Shanghai Institute (Shanghai, China). U‐87 MG cell lines were purchased from Beina Bio (Beijing, China). 1 × 10^6^ THP‐1 cells were treated with 100 ng/ml Phorbol‐12‐myristate‐13‐acetate (PMA, Sigma‐Aldrich) for 48h, and then added 25 ng/ml IL‐4 (Peprotech) for 48h to generate M2‐like macrophages for further research. The CD206 expression was evaluated with a FITC anti‐human CD206 (Biolegend, 321 103) by flowcytometry. The CD206 mRNA expression and PD‐L1 mRNA expression were detected using real‐time PCR.

### 2.5 SiRNA and real‐time PCR

The three human siRNA sequences were provided by Ribobio (Guangzhou, China). Seeding cells with 2 ml medium contain 1 μg siRNA and 2 μl Lipofectamine^TM^ 2000 (11,668,019; Thermo Fisher Scientific, USA) for 6 h at 37°C, then the medium was replaced with complete medium. The cells were collected after 48 h. Total RNA was extracted from U‐87 MG cell lines using TRIzol reagent (R1100, Solarbio, China) according to the manufacturer's instructions. After the gene‐silencing experiment, real‐time PCR was used to verify that LRRC15 gene product was successfully knocked down. The synthesis of cDNA was performed with PrimeScriptTM RT Master Mix Kit (RR036A; TAKARA, Tokyo, Japan). Real‐time PCR analysis was performed by TB Green® Premix Ex TaqTM II Kit (RR820A; TAKARA, Tokyo, Japan) in the Applied Biosystems 7300 Real‐Time PCR System (Applied Biosystems, Foster City, USA). The relative gene expression was calculated using the 2‐ΔΔCT method. The primer sequences were used as follows: LRRC15 (forward, 5′‐GCCTGTATGTACTGCTTTAACTC‐3′; reverse, 5′‐GGATAATGCCATTTCAGTGGT‐3′); CD206 (forward, 5′‐CTCTAAGCGCCATCTCCGTT‐3′; reverse, 5′‐ATGATCTGCGACTCCGACAC‐3′); PD‐L1 (forward, 5′‐TGGCATTTGCTGAACGCATTT‐3′; reverse, 5′‐TGCAGCCAGGTCTAATTGTTTT‐3′). The expression level of LRRC15, CD206, and PD‐L1 was standardized to GAPDH (Ribobio, China).

### 2.6 Transwell migration assay

The cell line U‐87 MG cells with or without LRRC15 knockdown were seeded on Day 0, and the culture supernatants were harvested on Day 8. Transwell inserts (Pore size 8.0 μm, Corning, 3422) were placed onto a 24‐well plate. A total of 500 μl of conditioned supernatant were applied to the bottom of per well. This was followed by the addition of 200 μl of THP1‐derived macrophages on the top of the inserts. After 48 h incubation at 37 ºC with 5% CO2, the insert membranes were stained with crystal violet, were dried and photographed underneath the membranes using a Nikon Eclipse TS100 Microscope (Nikon, Japan).

### 2.7 Cell proliferation by carboxy fluorescein succinimidyl ester (CFSE) tracking

The THP‐1 cells were seeded in transwell inserts (Pore size 0.4 μm, Corning, 3412) and were differentiated into M2‐like macrophages according to the above‐described method. The U‐87 MG cells with/without LRRC15 knockdown were labelled with carboxy fluorescein succinimidyl ester (CFSE, C375,DOJINDO) and were seeded into 6 well plates. Setting up the co‐culture system by combining the THP‐1‐derived M2‐like macrophages and CFSE‐labelled U‐87 MG cells with/without LRRC15 knockdown on the same plate. On d 4, U‐87 MG cells were obtained and the level of cell proliferation was tested by flow cytometry.

### 2.8 Statistical analysis

All data were expressed as mean ± SD (standard deviation). The Kaplan‐Meier survival curves were tested by the log‐rank test. Differential analysis of expressed genes and function analysis was performed using R version 3.5. SPSS (SPSS, Inc) software was used for statistical analysis. Wilcoxon rank‐sum test was used in groups with the non‐normally distributed variable. The independent‐samples t test was used in groups with a continuous variable. *P* < 0.05 was considered statistically significant in all tests.

## 3 RESULTS

### 3.1 Mining microenvironment‐associated genes in rGBM microenvironment

**FIGURE 1:**
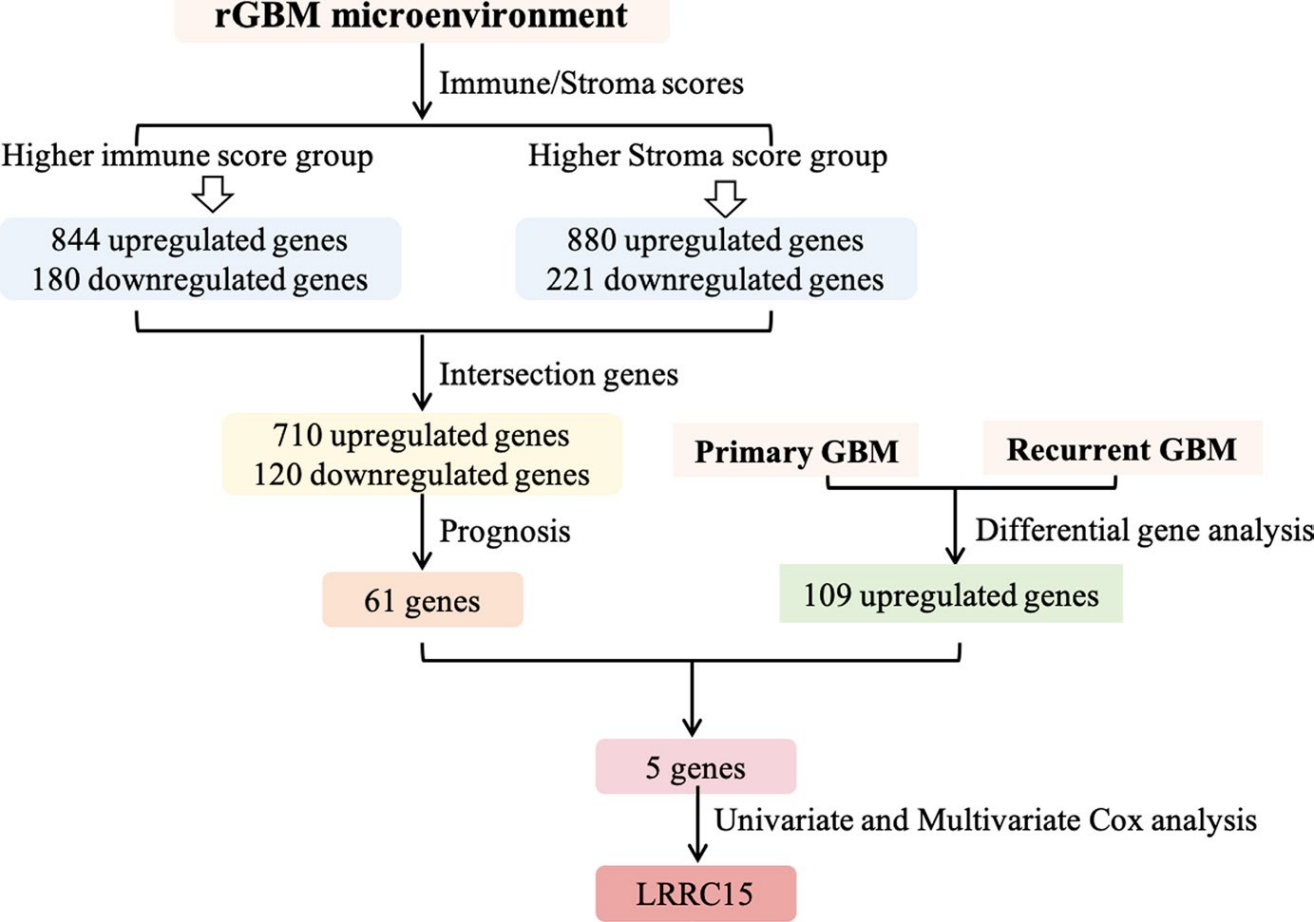
Schematic diagram showing the design of the study

**FIGURE 2:**
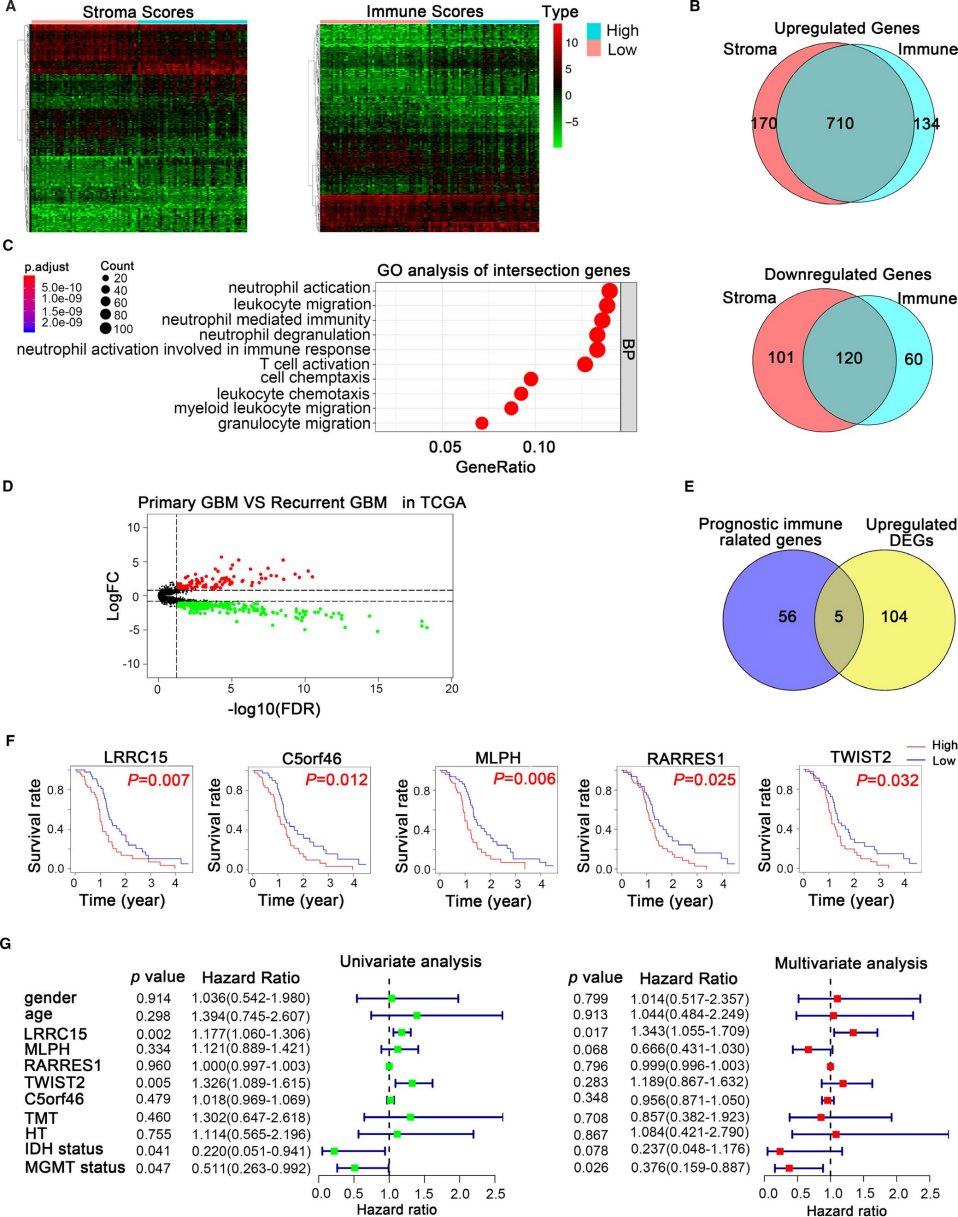
Prognostic immune‐associated genes of recurrent glioblastoma from TCGA database. (A) Heatmap of differential gene profiles (Top 100) based on immune scores and stroma scores. *P* < 0.05, fold change >1. (B) Venn diagrams showing the number of up‐regulated or down‐regulated interaction genes in immune score groups and stroma score groups. (C) GO analysis of interaction genes. GO, Gene Ontology; BP, Biological process (D) Volcanic plot visualizing the DEGs between primary and recurrent glioblastoma. DEGs, differentially expressed genes. *P* < 0.05, fold change >1. (E) Venn diagrams showing the prognostic interaction genes between up‐regulated immune‐associated genes and DEGs. (F) Kaplan‐Meier survival curves for the association of the five genes expression with OS in rGBM patients. *P* < 0.05 in log‐rank tests. (G) Univariate and multivariate Cox regression analysis explored the correlation between the gender, age, LRRC15, MLPH, RARRES1, TWIST2, C5orf46, TMT, HT, IDH status, and MGMT protomer status, and the OS. TMT, targeted molecular therapy; HT, hormone therapy; MGMT, methylation status of the O^6^‐methylguanine‐DNA methyl‐transferase (MGMT); OS, overall survival

We set out to mine genetic profiles that correlate with tumour microenvironment based on immune scores and stromal scores in rGBM (Figure 1). We performed RNA‐seq data of 169 recurrent GBM samples culled from TCGA. There were 844 up‐regulated genes and 180 down‐regulated genes in higher immune scores groups by comparing high with low scores groups (|log2 (fold change)| >1, *P* < 0.05). Similarly, 880 up‐regulated genes and 221 down‐regulated genes were obtained in higher stromal scores groups (|log2 (fold change)| >1, *P* < 0.05). Besides, the intersection genes included 710 up‐regulated and 120 down‐regulated in higher scores groups (Figure 2A, B). It is worth mentioning that these intersection genes were more closely correlated with tumour purity and played decisive roles in the TME. Furthermore, we identified the potential functional annotation of intersection genes based on Gene Ontology (GO) terms (false discovery rate, or FDR < 0.05, ‐log FDR >1.301). The results demonstrated that the top terms were 0042119‐neutrophil activation, 0031012‐extracellular matrix and 0048018‐receptor ligand activity (Figure 2C, Figure S1A), which suggested enrichment focussed on microenvironment‐associated pathways. To further identify the prognostic relevance of these intersection genes in rGBM patients, Kaplan‐Meier survival curves were generated to assess the clinical outcomes of the corresponding genes. The patient overall survival (OS) was assessed based on these genes’ expression, and we identified 61 genes potentially correlated with prognosis among 830 intersection genes using a log‐rank test (*P* < 0.05, Table 1). To reveal biomarkers can express differentially in the rGBM, we analysed DEGs between recurrent and primary GBM using TCGA RNA‐seq datasets, in which 109 up‐regulated genes and 209 down‐regulated genes were identified (|log2 (fold change)| >1, *P* < 0.05, Figure 2D). After that, we integrated these up‐regulated DEGs and the above‐identified prognosis‐associated markers to identify distinctive tumour‐specific immune‐associated biomarkers further. We identified five up‐regulated genes (LRRC15, C5orf46, MLPH, RARRES1 and TWIST2) in rGBM (Figure 2E, F). Notably, we further estimated the prognostic value of the five genes in rGBM patients concerning pharmacological therapy, including hormone therapy and targeted molecular therapy, showing high expression of LRRC15 and RARRES1 contributed to poor prognosis in patients without hormone, C5orf46, RARRES1, TWIST2 promoted poor prognosis in patients without targeted molecular therapy (Figure S1B‐E). Furthermore, we performed cox progression analysis to find key genes that could be used as independent risk factors for rGBM prognostic prediction. The univariate Cox progression analysis was used by integrating the mRNA expression profiles and clinical information of the five genes. LRRC15, TWIST2, IDH status and MGMT status were proved to be important predictors for patients with rGBM (*P* < 0.05). A multivariate Cox progression analysis further indicated that LRRC15 expression and MGMT status (*P* < 0.05) were independent risk factors for rGBM prognostic prediction. Therefore, we selected LRRC15 with significant differences in univariate and multivariate cox analysis for further analysis (Figure 2G). The univariate and multivariate Cox progression analysis demonstrated that LRRC15 can be used as an independent risk factor for rGBM prognostic prediction (Figure 2G). These results indicated that LRRC15 might be a potential rGBM target for the development of novel therapeutic strategies.

**TABLE 1:** The genes with prognostic value

gene	median survival time (low/high)	*P‐value*
CTHRC1	543/405	0.04640013
ENTHD1	480/399	0.03209777
LOXL2	543/395	0.04712455
THBD	485/399	0.03610763
RARRES1	535/405	0.02545571
MMP1	485/405	0.03576519
SOCS3	489/399	0.01850587
LSP1	505/385	0.02705352
FN1	505/375	0.01360658
COL6A3	489/385	0.04952642
FOSL1	543/394	0.00592895
MMP19	505/399	0.04172311
NEU4	399/535	0.01619315
ELF5	489/427	0.03177262
STC1	532/360	0.0000505
BDKRB2	548/394	0.02712349
C5orf46	489/399	0.01214094
KRT80	548/394	0.01665271
TMEM26	485/399	0.03432677
SLC17A9	532/385	0.03520527
ADAMTS14	532/399	0.00367251
CXCL5	505/394	0.00539419
ANKRD1	598/405	0.01065263
BAIAP2L1	535/399	0.00983912
COL5A1	489/399	0.0407329
MXRA5	532/399	0.00927983
VDR	489/385	0.02797673
LRRC15	505/362	0.00724822
TFPI2	489/405	0.03295255
THBS1	489/399	0.01906768
ADAM8	485/442	0.02581667
TPSAB1	489/375	0.04994894
INHBA	543/399	0.03764112
ST8SIA5	532/448	0.03742567
TWIST2	489/405	0.03159857
AQP9	485/394	0.03648032
FAM81B	480/448	0.03524028
RFX8	532/399	0.00599177
FAM20A	505/399	0.02453916
TMEM200B	505/399	0.02056244
IL11	505/394	0.02994168
AKR1B15	406/543	0.02706454
PLBD1	489/394	0.04990027
SCG3	399/535	0.00805953
FOLR2	442/505	0.02990354
PCOLCE	532/394	0.02753339
CCR7	532/385	0.02048157
ARSI	603/406	0.02692185
COL1A1	489/394	0.03218838
ANKEF1	548/385	0.00132887
MLPH	532/394	0.0055648
SLAMF8	505/399	0.04185203
ORM1	489/405	0.02143096
CDCP1	505/375	0.03704625
HMGA2	548/394	0.00099434
TGFBI	485/399	0.03234585
IL7R	489/442	0.03306669
RETN	505/399	0.00718056
HES5	399/489	0.04545499
COL6A2	543/399	0.01711164
MMP11	548/399	0.00955751

### 3.2 Investigation of potential correlation of LRRC15 gene with infiltration of GAMs in rGBM

**FIGURE 3:**
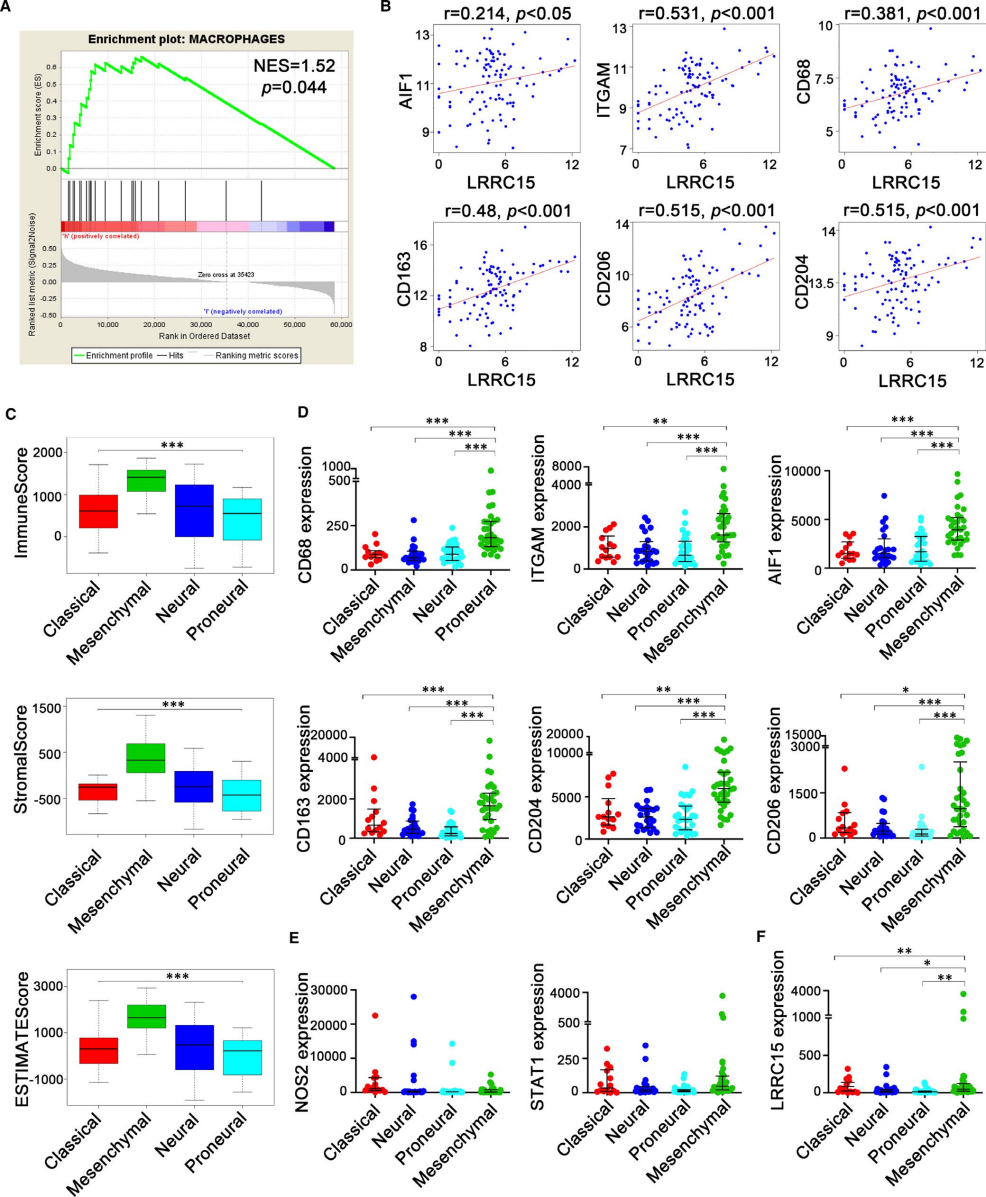
Association and transcriptional subtypes heterogeneity between LRRC15 expression and GAMs. (A) Positive correlation between LRRC15 and macrophages representative gene. Twenty‐two genes were selected as macrophages representative genes for the gene set enrichment^26^ NES, normalized enrichment score; FWER *P*‐val, family‐wise error rate *P*‐value. (B) Correlation of LRRC15 and respective markers of microglia/macrophages from TCGA RNA‐seq data. (C) Differences of tumour purity among subtypes were shown with distribution of immune scores, stromal scores and estimate scores. (D, E) Expression of phenotypic surface markers of microglia/macrophages in subtypes. (F) LRRC15 expression levels in the mesenchymal GBM subtype were higher than other GBM subtypes. The gene expression level was defined as RSEM (RNA‐seq by expectation‐maximization), and the patient's information was culled from TCGA RNA‐seq datasets (n = 101). Significant differences are indicated by asterisks per the ANOVA with the Holm test, with bars indicating SD (*P < 0.05, **P < 0.01, ***P < 0.001)

We aimed to elucidate whether the expression of LRRC15 might involve in infiltration of GAMs in rGBM. For this purpose, we analysed the potential correlation between macrophages and LRRC15 expression in rGBM. The results demonstrated that LRRC15 expression was indeed correlated with the macrophage gene set^26^ (Figure 3A). We further explored the relationship between LRRC15 expression and microglia/macrophages markers levels using TCGA database RNA‐seq data. We found the significant association between high LRRC15 expression and the high level of integrin subunit alpha M (ITGAM, also known as CD11B), allograft inflammatory factor 1 (AIF1, also known as IBA1) and CD68. In particular, LRRC15 expression was also significantly positively correlated with transcriptional levels of M2‐like macrophages markers CD206, CD163 and CD204 but was not positively related to M1‐like macrophages markers NOS2 and STAT1 (Figure 3B, Figure S2A, B).

Notably, we quantified the tumour purity of rGBM molecular subtypes using ESTIMATE method. The results showed the mesenchymal subtype had the highest immune and stromal scores (Figure 3C). To define the incidence of microglia/macrophages in distinct rGBM subtypes, we analysed the above‐described markers using TCGA database. The results showed markedly higher expression of ITGAM, AIF1, CD206, CD163 and CD204, but not NOS2 and STAT1, in mesenchymal compared with non‐mesenchymal subtypes (Figure 3D, E). Next, to get insight into LRRC15 expression in subtypes, we interrogated the expression of LRRC15 in the different subtypes using TCGA database. Interestingly, the results demonstrated LRRC15 was remarkably high expressed in the mesenchymal subtype (Figure 3F). These data demonstrated that mesenchymal subtypes are better candidates for targeting LRRC15 therapies against GAMs.

### 3.3 Validation in CGGA database

**FIGURE 4:**
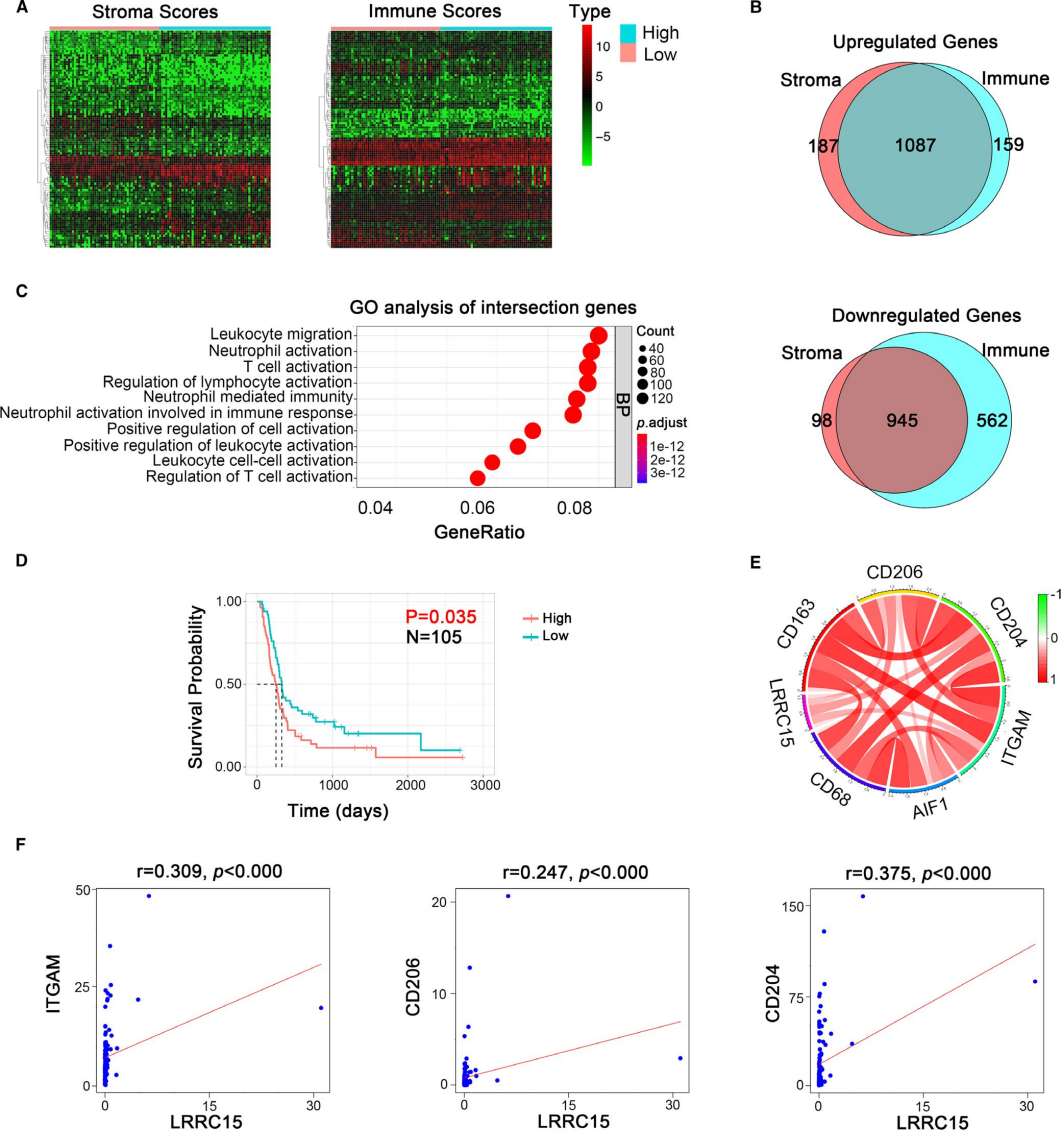
Validation of immune association of LRRC15 in the CGGA data set. (A) Heatmap of differential gene profiles (Top 50) based on immune scores and stroma scores. *P* <0.05, fold change >1, including LRRC15. (B) Venn diagrams showing the number of up‐regulated or down‐regulated interaction genes in immune score groups and stroma score groups. (C) GO analysis of interaction genes. GO, Gene Ontology. BP, Biological process (D) Kaplan‐Meier survival curves for the association of LRRC15 expression with overall survival (OS) in rGBM patients. *P* < 0.05 in log‐rank tests. (E‐F) Validation of correlation of LRRC15 and respective markers of microglia/macrophages from CGGA RNA‐seq data

To validate the immune association and prognostic value of LRRC15, we downloaded mRNAseq_693 from CGGA database.^21^, ^22^ We chose 109 rGBM cases to quantify TME using the ESTIMATE method. The results demonstrated 1087 up‐regulated genes and 945 down‐regulated genes in the intersection genes between higher immune score groups and higher stroma score groups (Figure 4A, B). Similarly, we also identified that the intersection genes could enrich in the microenvironment‐associated pathways based on Gene Ontology (GO) terms (false discovery rate, or FDR < 0.05, ‐log FDR >1.301) (Figure 4C). Fortunately, we found LRRC15 existed in up‐regulated intersection genes (Table S1). Furthermore, we verified the correlation of LRRC15 expression with prognostic value in rGBM (Figure 4D). Meanwhile, we confirmed the correlation between LRRC15 expression and macrophages transcriptional levels in the rGBM cases (Figure 4E, F). Besides, the gene profiles from Chinese Glioma Genome Atlas (CGGA) data portal (mRNAseq_325) were collected. The immune association of LRRC15 in rGBM was further validated based on ESTIMATE algorithm. We found 599 up‐regulated genes and 122 down‐regulated genes in the intersection genes between higher immune score groups and higher stroma score groups (Figure S2C, D). Meanwhile, the intersection genes could enrich in the immune‐associated pathways based on Gene Ontology (GO) terms (false discovery rate, or FDR <0.05, ‐log FDR >1.301) (Figure S2E). Similarly, LRRC15 also existed in up‐regulated interaction genes (Table S2). Furthermore, LRRC15 expression is enriched in macrophage gene set in rGBM (Figure S2F). These results strengthened the characteristics of LRRC15 correlated with poor prognosis and GAMs infiltration in rGBM.

### 3.4 Confirmation of LRRC15 expression promoting macrophages infiltration and tumour poor prognosis

**FIGURE 5:**
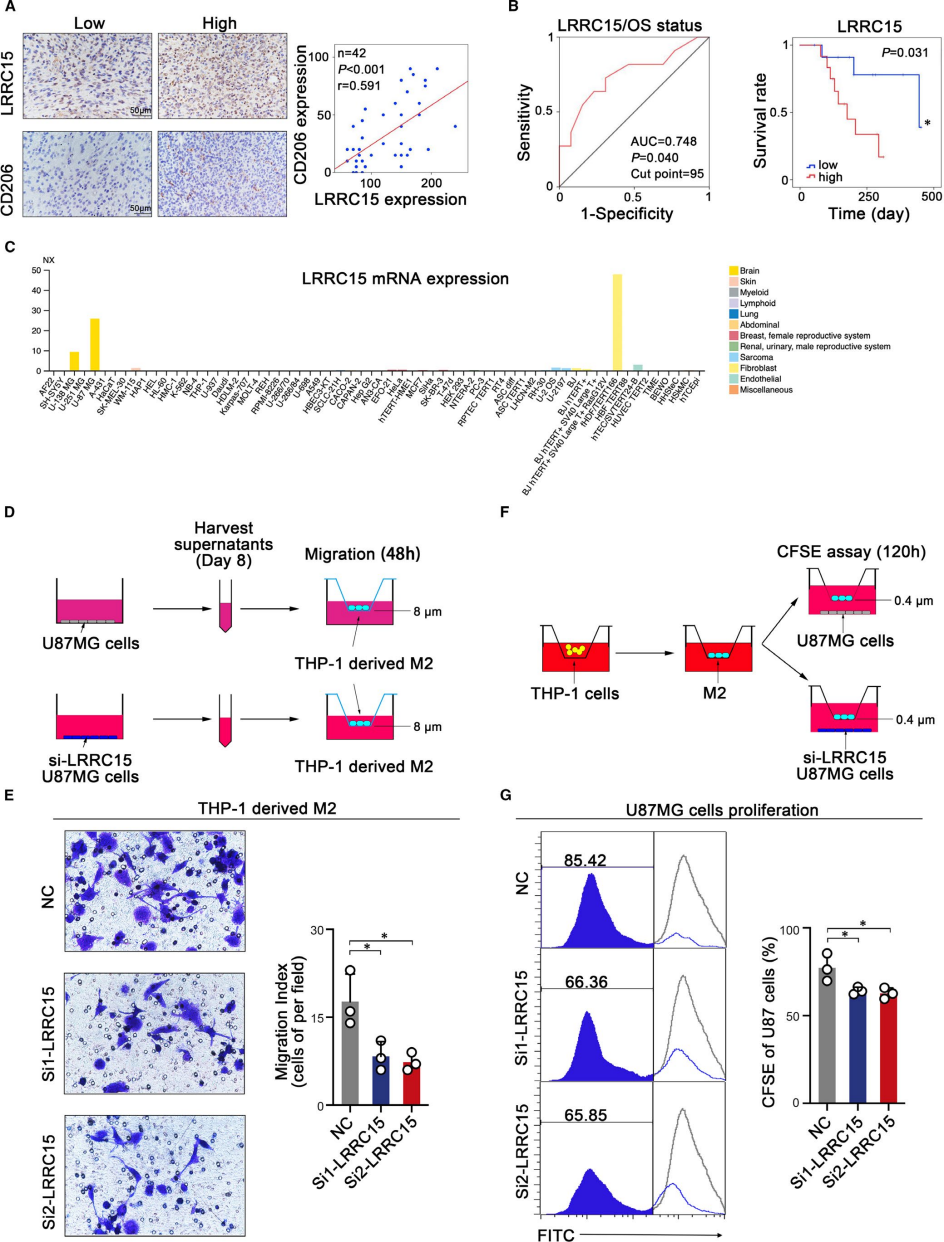
LRRC15 promotes the recruitment of GAMs and proliferation of tumour cells. (A) Representative micrographs of LRRC15 expression and CD206 expression as well as their expression correlation analysis in human rGBM tissues by immunohistochemistry assay. (B) Kaplan‐Meier curve showed the association between LRRC15 expression and OS in 24 rGBM patients with survival information based on the relative LRRC15 IHC score divided by the optimal cut‐point off. ROC curve analysis of LRRC15 IHC score was shown based on OS status. (C) LRRC15 expression in multiple cells from Human Protein Atlas. (D) Schematic diagram of experimental design of tumour LRRC15 expression influencing M2‐like macrophage migration. Supernatants of tumour cell culture were collected on Day 8 after the tumours were seeded and used for the M2‐like macrophages migration assay. (E) Migration analysis of M2‐like macrophages in the presence of LRRC15 or LRRC15 knockdown tumour supernatants by transwell assay. Images were taken 48 h after the M2‐like macrophages were exposed to the tumour supernatants. The images represent 1 of 3 individual experiments using THP‐1‐derived macrophages. (F) Schematic diagram of experimental design of tumour LRRC15 expression influencing tumour proliferation in the presence of M2‐like macrophages. THP‐1 cells were induced into M2‐like macrophages and co‐cultured with U‐87 MG cells with/without LRRC15 knockdown for CFSE assay (G) CFSE assay was used to examine the tumour cells proliferation with/without LRRC15 knockdown

In order to further confirm the distinct characteristics of LRRC15 in rGBM, we performed immunohistochemistry assay and Kaplan‐Meier survival analysis in clinical samples of rGBM after validation with TCGA database and CGGA data sets. The IHC assay results showed LRRC15 expression was positively correlated with CD206 expression (Figure 5A). Meanwhile, the Kaplan‐Meier survival analysis suggested LRRC15 high expression promoted poor prognosis of rGBM patients (Figure 5B). Furthermore, tumour cells have been shown to recruit macrophages.^27^ We used the Human Protein Atlas (https://www.proteinatlas.org) to analyse the LRRC15 mRNA level in multiple cell lines, and LRRC15 expression showed enhancement in U‐87 MG cells (Figure 5C), which made U‐87 MG cell line priority. Based on the above‐described bioinformatic analysis of the significant correlation between LRRC15 and M2‐like macrophages, we sought to confirm the direct connection exists between LRRC15 and M2‐like macrophages. The THP‐1‐derived M2‐like macrophages were identified using flowcytometry and real‐time PCR (Figure S3). Furthermore, we performed the transwell assay using supernatants derived from GBM cell line U‐87 MG with/without LRRC15 knockdown co‐cultured with THP‐1‐derived M2‐like macrophages (Figure 5D). The results showed that the supernatants from LRRC15 knockdown U‐87 MG cells recruited less M2‐like macrophages as compared with the NC group (Figure 5E). Furthermore, to prove the correlation of LRRC15 and the poor prognosis of rGBM, we simulated the TME in a co‐culture system to perform the CFSE assay (Figure 5F). The results showed LRRC15 knockdown could inhibit the proliferation of U‐87 MG cells compared with the NC group in the presence of M2‐like macrophages (Figure 5G). These results from the in vitro experiments suggest that LRRC15 is responsible, at least in part, for the attraction of GAM and proliferation of tumour cells.

## 4 DISCUSSION

Recent breakthroughs that use checkpoint inhibitors have been successful in some tumours,^28^, ^29^, ^30^ while their clinical benefits were limited in GBM (such as the CheckMate‐143),^8^ partly because of the low tumour mutation burden (TMB) of GBM intrinsic characteristics^31^, ^32^ and immunosuppressive microenvironment.^10^, ^33^ Tumour cells, stromal cells and immune cells shaped distinct tumour microenvironment. The lower TMB had lower tumour purity, which indicated understanding rGBM microenvironment‐associated contents and identifying closely related biomarkers responsible for immunosuppression in GBM are critical in the development of successful immunotherapeutic strategies. In the present study, we analysed the genomic signatures of rGBM microenvironment based on ESTIMATE algorithm, which has been optimized and applied to many tumours to estimate tumour purity.^4^, ^34^ By combining the up‐regulated DEGs between primary and recurrent GBM with TME‐related genes, we further identified the five specific rGBM‐associated genes: LRRC15, C5orf46, MLPH, RARRES1 and TWIST2, which were related to patients' prognosis of rGBM. Interestingly, using univariate and multivariate analysis, we not only confirmed the positive prognostic role of MGMT status as an independent prognostic factor, which was supported by some strong evidence,^35^, ^36^ but also confirmed the negative survival role of LRRC15 as an independent prognostic factor, which indicated that LRRC15 could be used as a novel biomarker for predicting the rGBM patients’ outcomes. In particular, the low expression of LRRC15 in patients without hormone therapy was associated with good clinical outcomes.

Tumour‐associated macrophages could contribute to poor prognosis in solid tumours.^37^, ^38^, ^39^ GAMs predominate the tumour microenvironment in GBM.^40^ The infiltration of GAMs could mediate radio‐/chemotherapy tolerance, angiogenesis and metastasis of GBM,^4^, ^41^ indicating GAMs are important parameters to consider in developing immunotherapeutic strategies of GBM. Gene Ontology (GO) analysis of the study also validated that these TME‐associated genes are involved in leucocyte migration in rGBM. Therefore, we further investigated the correlation between LRRC15 and macrophages infiltration. By using an established immune gene signatures approach,^26^ the GSEA gene set enrichment uncovered the association between LRRC15 expression and macrophages in rGBM. In the brain, macrophages derived from brain‐resident microglia/macrophages and peripheral monocytes/macrophages.^9^, ^10^ The further study employed transcriptional levels of the representative macrophages/microglial markers^4^, ^41^, ^42^ to identify their relationship with LRRC15 expression. Fortunately, the LRRC15 expression was positively correlated with the expression of ITGAM, AIF1, CD68, CD206, CD163 and CD204 with M2‐like phenotype and was not with NOS2 and STAT1 represented the M1‐like functional phenotype.^43^, ^44^ Furthermore, the CGGA database, an independent database, was used as validation (including two data sets). Importantly, our IHC assay also confirmed the LRRC15 expression promoted the poor prognosis and positively associated with CD206 expression in our rGBM clinical samples. The study also confirmed LRRC15 could contribute to GAMs infiltration and tumour proliferation in GBM, which was consistent with previous studies that many LRR‐containing proteins are involved in tumour progression.^45^, ^46^, ^47^ Meanwhile, an LRRC15‐targeting antibody‐drug conjugate, ABBV‐085, is currently being evaluated in a Phase 1 safety study.^20^ Our results indicated LRRC15 may be an underlying target to harness GAMs biology to affect prognosis for rGBM patients. Furthermore, GAMs could promote immunosuppression through multiple mechanisms in the TME including the up‐regulation of PD‐L1 and B7‐H1.^48^, ^49^ Dominguez et al suggested LRRC15^+^ carcinoma‐associated fibroblasts are associated with poor anti‐PD‐L1 immunotherapy response.^50^ Thus, the strategies of targeting LRRC15 to ablate GAMs effects in the rGBM microenvironment could improve effects of immune‐checkpoint inhibitors.

Extensive studies also highlighted the heterogeneity of molecular subtypes of GBM in TME to understand the complexity of GBM.^15^, ^16^, ^51^ GBM demonstrated considerable heterogeneity of clinical response for GBM molecular subtypes to current treatments. The molecular subtype heterogeneity is associated with the TME, and the subtypes did not change at recurrence.^51^ Therefore, understanding molecular subtype of rGBM is capable of providing better predictions of rGBM progression and treatment choices. We qualified the tumour purity of different subtypes of rGBM, and the mesenchymal subtype showed the lowest tumour purity, which was consistent with previous studies indications of the expansion of microglia/macrophages in the mesenchymal subtype.^42^, ^52^, ^53^ Our qualified molecular subtypes of rGBM also demonstrated the correlation between mesenchymal subtype with increased levels of M2‐like macrophages. The results implied that the mesenchymal subtype could enhance the responsiveness of macrophages‐based therapeutic strategies. Interestingly, the expression of LRRC15 showed a significant increase in the mesenchymal subtype, suggesting targeting LRRC15 could be more beneficial for rGBM treatments.

In summary, the study comprehensively analysed the rGBM microenvironment gene signatures, and integrated rGBM microenvironment‐associated genes and up‐DEGs in rGBM to identify a novel prognostic immune‐related gene, LRRC15. Our results confirmed that the high expression of LRRC15 was associated with the infiltration of GAMs. Meanwhile, we also identified LRRC15 expression was enriched in mesenchymal subtype with GAMs‐rich microenvironment. Our results may aid to interpret the rGBM microenvironment and to provide a novel therapeutic target to improve rGBM prognosis.

## CONFLICT OF INTEREST

The authors declared that they have no conflicts of interest to this work.

## AUTHOR CONTRIBUTIONS

**Haichao Tang:** Conceptualization (equal); Data curation (lead); Formal analysis (lead); Methodology (equal); Writing‐original draft (lead). **Wensi Liu:** Conceptualization (equal); Formal analysis (equal); Investigation (equal). **Zhaoxu Xu:** Data curation (supporting); Formal analysis (equal). **Jianhang Zhao:** Formal analysis (supporting); Methodology (supporting); Visualization (supporting). **Weitao Wang:** Data curation (supporting); Validation (supporting). **Zhaojin Yu:** Conceptualization (equal); Data curation (equal); Formal analysis (equal); Supervision (lead); Writing‐original draft (supporting). **Minjie Wei:** Conceptualization (lead); Funding acquisition (lead); Validation (equal); Writing‐original draft (equal).

## Supporting information

Fig S1‐S3

Table S1

Table S2

## Data Availability

The data that support the findings of this study are available from the corresponding author upon reasonable request.
