# Supplementary material for: Integrated microenvironment‐associated genomic profiles identify LRRC15 mediating recurrent glioblastoma‐associated macrophages infiltration
Source: J Cell Mol Med. 2021 May 7;25(12):5534–46. doi: 10.1111/jcmm.16563 (PMC8184692; doi:10.1111/jcmm.16563)
Supplement: Supplementary file 1 — Fig S1‐S3 [file JCMM-25-5534-s003.pdf]

Figure S1.

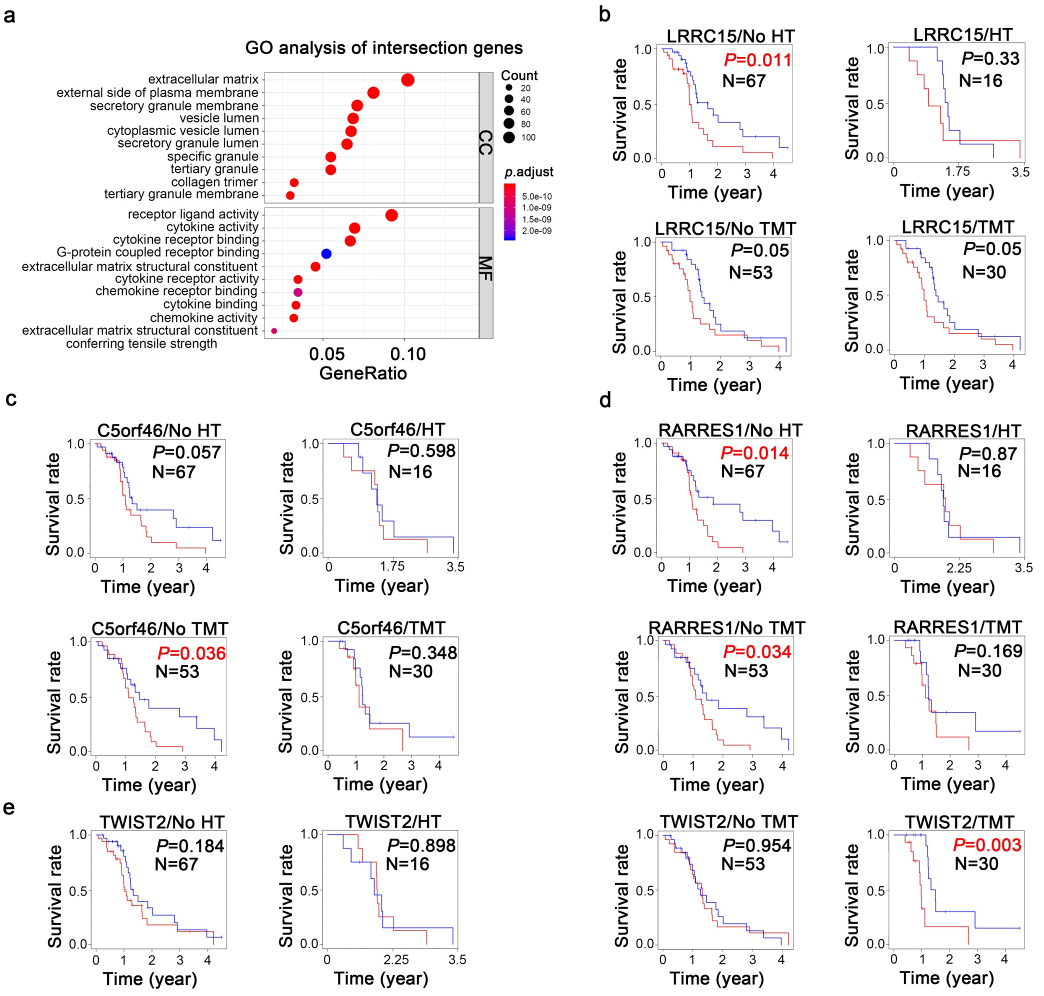

Figure S1. Prognostic value of immune-associated genes. (a) GO analysis of interaction genes. GO, Gene Ontology. CC, Cellular component, MF, Molecular function. (b-e) Kaplan-Meier survival curves for estimating prognostic value of LRRC15, C5orf46, MLPH, RARRES1, and TWIST2 of rGBM patients in different pharmaceutical therapies. HT, hormone therapy; TMT, targeted molecular therapy. P <0.05 in log-rank tests.

Figure S2

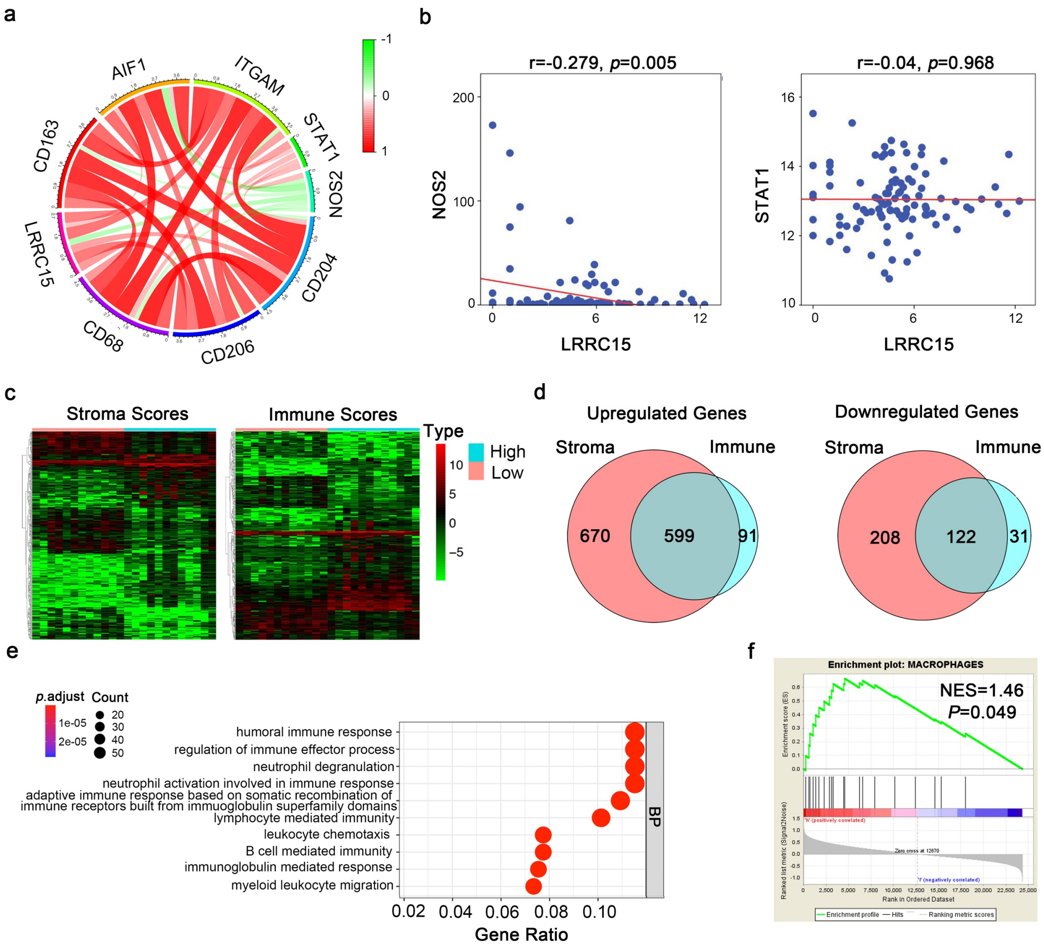

Figure S2. Correlation between LRRC15 and macrophages. (a) Association of LRRC15 and microglia/macrophages from TCGA RNA-seq data. (b) Correlation of LRRC15 and respective markers of M1-like macrophages from TCGA RNA-seq data. (c) Heatmap of differential genes (Top 200) based on immune and stroma scores from CGGA dataset (mRNAseq_325). *P* <0.05, fold change >2, including LRRC15. (d) The number of interaction genes in immune score group and stroma score group were showed using Venn diagrams. (e) GO analysis of the interaction genes. (f) Validation of correlation of LRRC15 and macrophages using GSEA gene set enrichment.

Figure S3

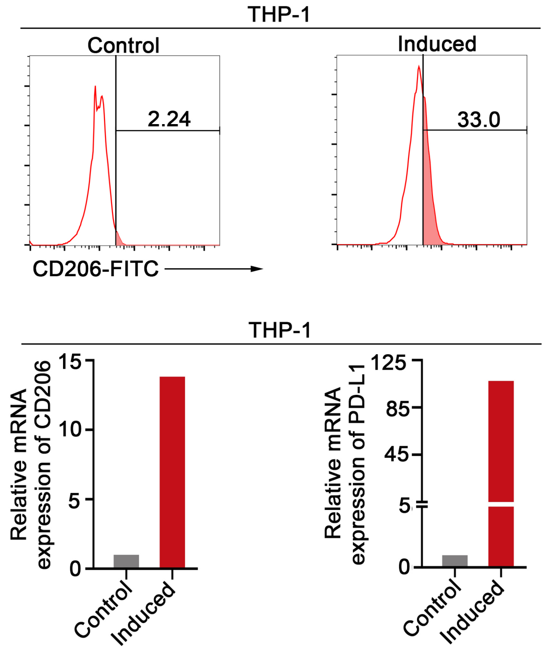

Figure S3. **The phenotype assay of THP-1 induced M2 macrophages by CD206 expression using flowcytometry and CD206 mRNA as well as PD-L1 mRNA expression using real-time PCR.**
